# Supplementary material for: Crustal permeability generated through microearthquakes is constrained by seismic moment
Source: Nat Commun. 2024 Mar 6;15:2057. doi: 10.1038/s41467-024-46238-3 (PMC10918097; doi:10.1038/s41467-024-46238-3)
Supplement: Supplementary file 1 — Supplementary Information [file 41467_2024_46238_MOESM1_ESM.pdf]

Supplementary Information for "Crustal Permeability Generated through Microearthquakes is Constrained by Seismic Moment"

Pengliang Yu[1], Ankur Mali[2], Thejasvi Velaga[3], Alex Bi[4], Jiayi Yu[1], Chris Marone[1,5], Parisa Shokouhi[6], Derek Elsworth[1]

[1]EMS Energy Institute and Department of Geoscience, Pennsylvania State University, University Park, United States
[2]Department of Computer Science & Engineering, University of South Florida, Tampa, FL, United States
[3]Department of Computer Science and Engineering, Pennsylvania State University, University Park, PA, United States
[4]Pennsylvania State University, University Park, PA, United States
[5]Dipartimento di Scienze della Terra, La Sapienza Università di Roma, Roma, Italy
[6]Department of Engineering Science and Mechanics, Pennsylvania State University, University Park, PA, United States

Content of this file

1. Figure S1: Boxplots of seismicity rate and cumulative log seismic moment features of EGS Collab and Utah FORGE datasets.
2. Figure S2: Normalized permeability change $\Delta(k/k_0)$ versus seismic moment (first row) and normalized permeability (k/k_0) versus cumulative seismic moment (second row) for stimulation episode 3 (Ep3: A1, B1), episode 4 (Ep4: A2, B2) and episode 5 (Ep5: A3, B3) at EGS Collab.
3. Figure S3: Normalized permeability change $\Delta(k/k_0)$ versus seismic moment (first row) and normalized permeability (k/k_0) versus cumulative seismic moment (second row) for stimulation Stage 1 (A1, B1), Stage 2 (A2, B2) and Stage 3 (A3, B3) at Utah FORGE.
4. **Figure S4**: Seismicity rate changes over time under different Δt_w for EGS Collab dataset. With increases of Δt_w, the seismicity rate changes become much smoother for three episodes.
5. **Figure S5**: Seismicity rate changes over time under different Δt_w for Utah FORGE dataset. With increases of Δt_w, the seismicity rate changes become much smoother for three stages.
6. **Figure S6**: Comparison between raw permeability data (ground truth) and predictions on test set (Ep5) for EGS-Collab for different α values. With increasing α, the prediction curve on the test set monotonically increases. $\alpha = 1000$ is used in this study shown in Fig.6A of manuscript.

7. **Figure S7**: Comparison between raw permeability data (ground truth) and predictions on test set (S3) for Utah FORGE for different α values. With increasing α, the prediction curve on the test set monotonically increases. $\alpha = 150$ is used in this study shown in Fig.6B of manuscript.
8. **Figure S8**: Conceptual flow diagram for radial (a) and spherical (b) steady flow. Here r_w is the wellbore radius, r_t is the radius to the external flow boundary and h is the length of the borehole/cylindrical-zone.
9. **Figure S9:** Magnitude frequency distribution and Gutenberg-Richter fit with uncertainty analysis for EGS Collab (a) and Utah FORGE (b) datasets, respectively.
10. Table S1: Comparison of various standalone machine learning and deep learning models trained on Utah FORGE. All deep learning models were trained across 10 trials, with average R^2 scores and variances reported across data splits.
11. Table S2: Comparison of various standalone machine learning and deep learning models trained on EGS Collab. All deep learning models were trained across 10 trials, with average R^2 scores and variances reported across data splits.
12. Table S3: Zero-shot performance for models t**rained on EGS Collab and tested on Utah FORGE**. All deep learning models were trained across 10 trials, with average R^2 scores and variances reported across data splits.
13. **Table S4:** Zero-shot performance for models **trained on Utah FORGE and tested on EGS Collab**. All deep learning models were trained across 10 trials, with average R^2 scores and variances reported across data splits.
14. **Table S5:** Transfer learning performance for model trained on Utah FORGE and knowledge is transferred on EGS Collab. All deep learning models were trained across 10 trials, with average R^2 scores and variances reported across data splits.
15. **Table S6:** Transfer learning performance for models trained on EGS Collab and knowledge is transferred on Utah FORGE. All deep learning models were trained across 10 trials, with average R^2 scores and variances reported across data splits.
16. **Table S7:** Model performance comparison between models using proposed physics inspired loss and standard MSE loss (α = 0) for standalone models trained on Utah FORGE dataset. All deep learning models were trained across 10 trials, with average R^2 scores and variances reported across data splits.
17. **Table S8:** Model performance comparison between models using proposed physics inspired loss with standard MSE loss α = 0) for standalone models trained on EGS Collab dataset. All deep learning models were trained across 10 trials, with average R^2 scores and variances reported across data splits.
18. **Table S9:** Comparison of Bi-LSTM models using different values of Δt_w trained on EGS Collab. All models were trained across 10 trials, with average R^2 scores and variances reported across data splits.

19. Table S10: Comparison of Bi-LSTM models using different values of Δt_w trained on Utah FORGE. All models were trained across 10 trials, with average R^2 scores and variances reported across data splits.

20. **Table S11:** Comparison of LSTM and Bi-LSTM models trained on EGS collab. All models were trained across 10 trials, with average R^2 scores and variances reported on test set.

21. Table S12: Nomenclature

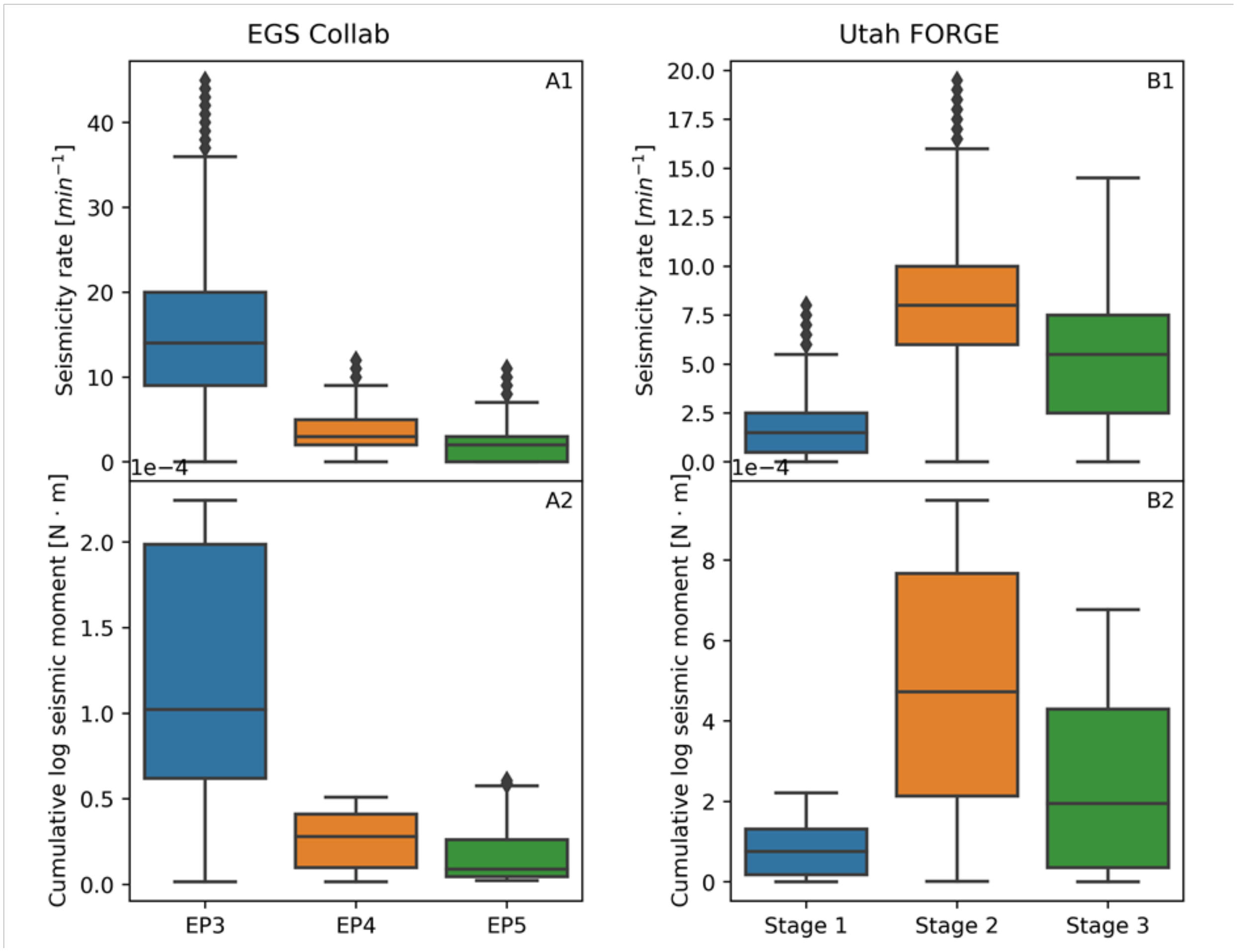

Figure S1: Boxplots of seismicity rate and cumulative log seismic moment features of EGS Collab and Utah FORGE datasets. The first column shows the different seismicity rate (A1), and cumulative log seismic moment (A2) distribution among EP3, EP4, and EP5 of EGS collab. The second column shows the different seismicity rate (B1), and cumulative log seismic moment (B2) among Stage 1, Stage 2, and Stage 3 of Utah FORGE dataset.

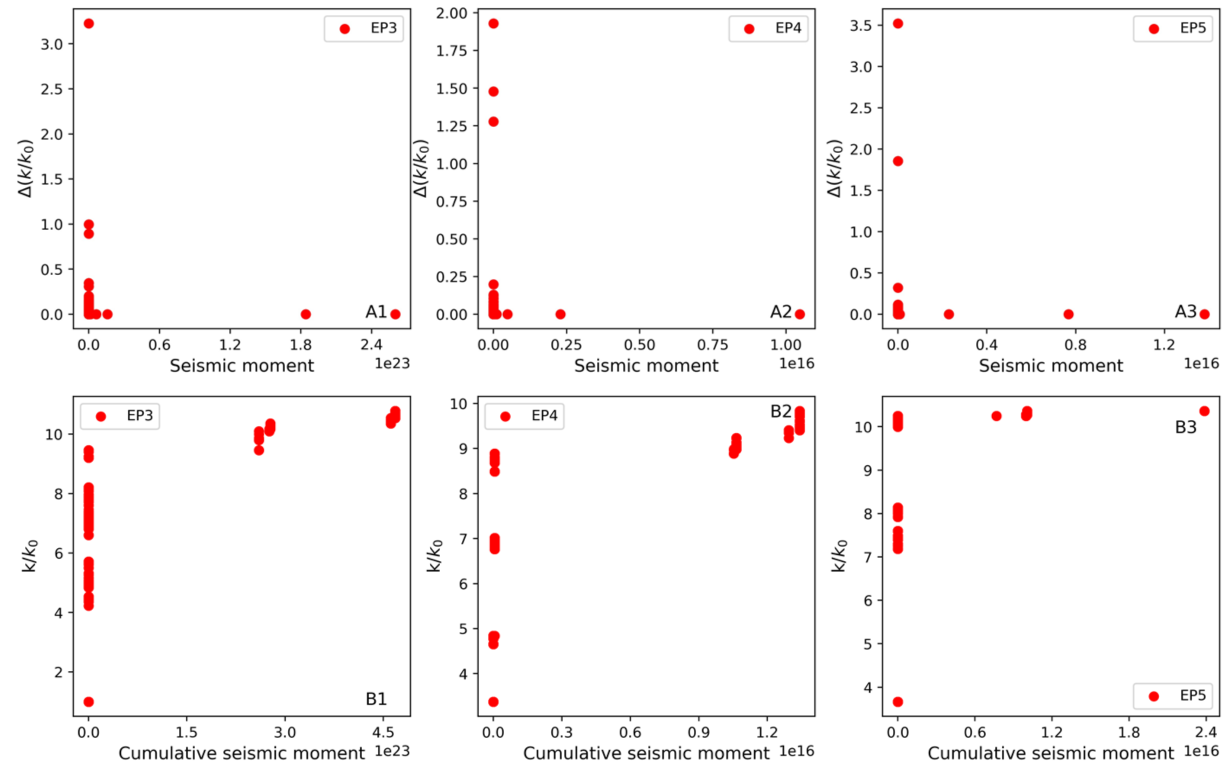

Figure S2: Normalized permeability change $\Delta(k/k_0)$ versus *seismic moment* (first row) and normalized permeability (k/k_0) versus *cumulative seismic moment* (second row) for stimulation episode 3 (Ep3: A1, B1), episode 4 (Ep4: A2, B2) and episode 5 (Ep5: A3, B3) at EGS Collab.

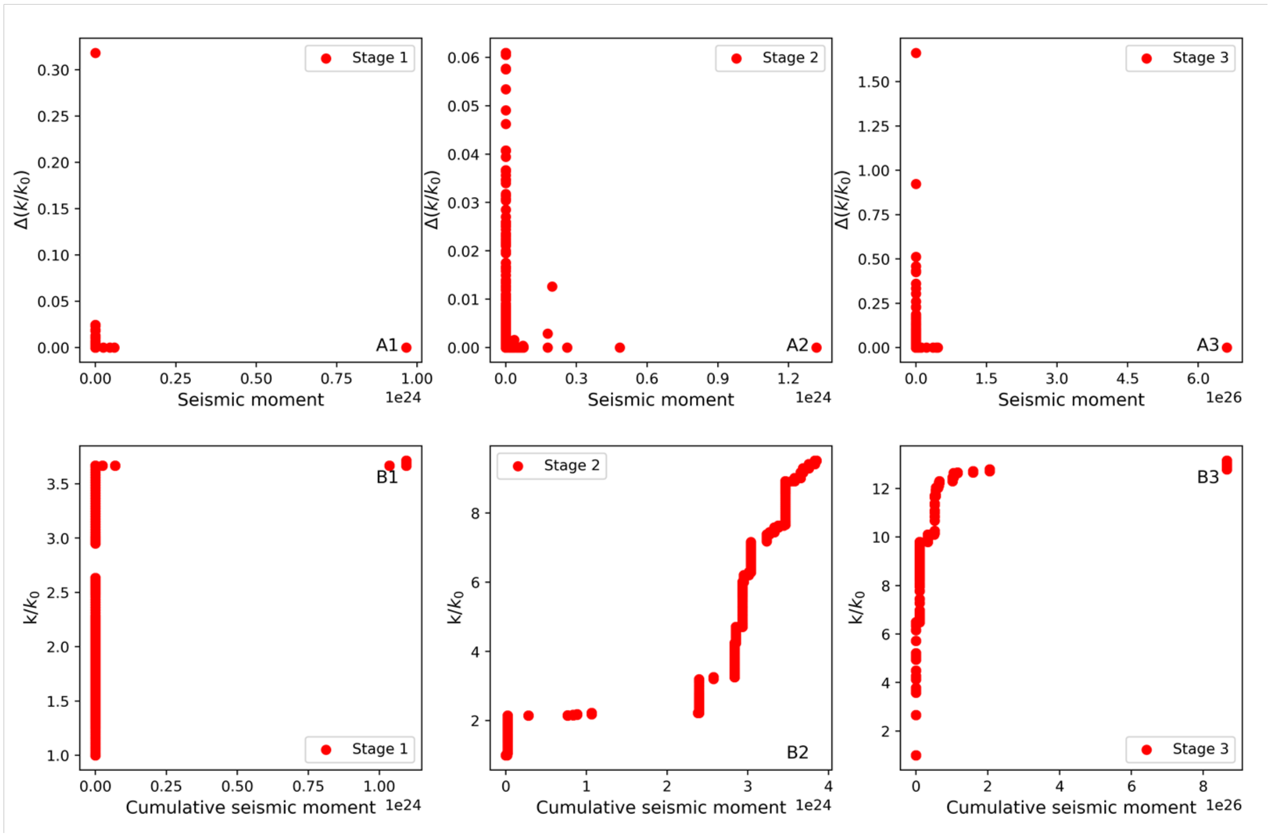

Figure S3: Normalized permeability change $\Delta(k/k_0)$ versus *seismic moment* (first row) and normalized permeability (k/k_0) versus *cumulative seismic moment* (second row) for stimulation Stage 1 (A1, B1), Stage 2 (A2, B2) and Stage 3 (A3, B3) at Utah FORGE.

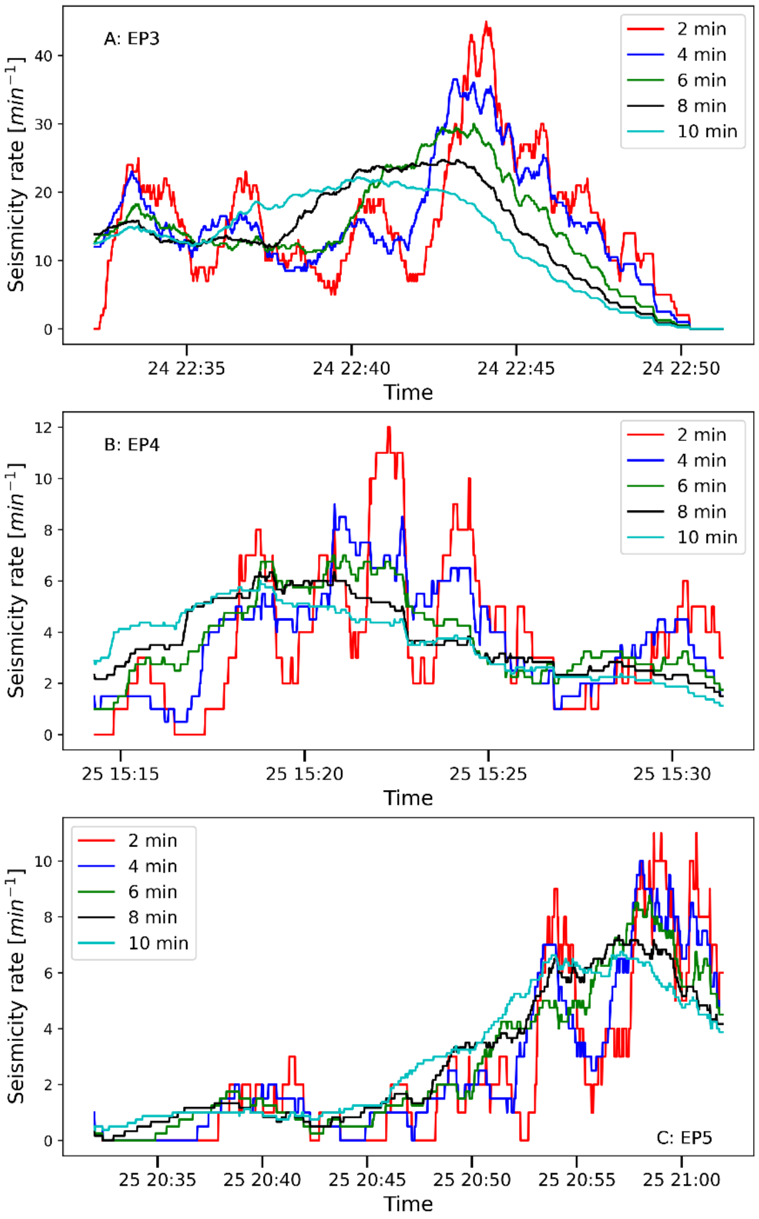

Figure S4: Seismicity rate changes over time under different Δt_w for EGS Collab dataset. With increases of Δt_w, the seismicity rate changes become much smoother for three episodes.

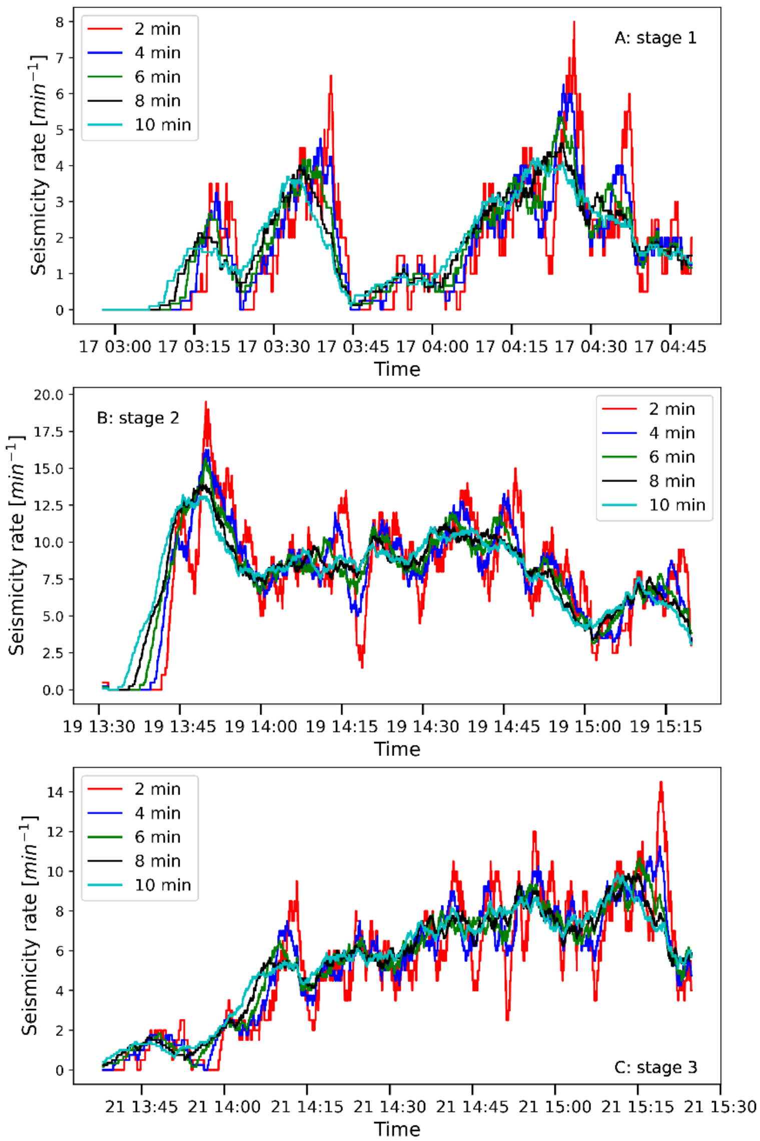

Figure S5: Seismicity rate changes over time under different Δt_w for Utah FORGE dataset. With increases of Δt_w, the seismicity rate changes become much smoother for three stages.

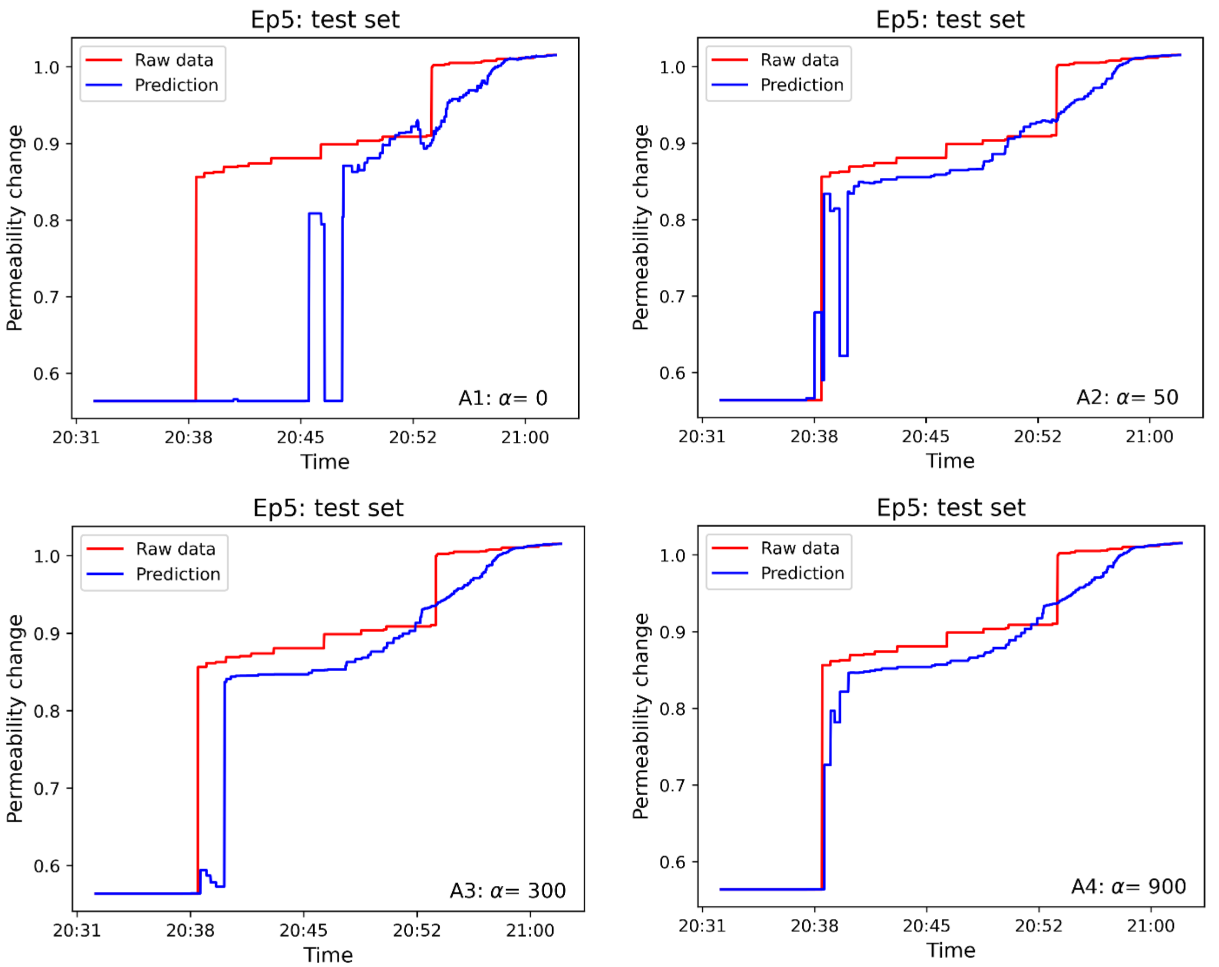

Figure S6: Comparison between raw permeability data (ground truth) and predictions on test set (Ep5) for EGS-Collab for different α values. With increasing α, the prediction curve on the test set monotonically increases. $\alpha = 1000$ is used in this study shown in Fig. 6A of the manuscript.

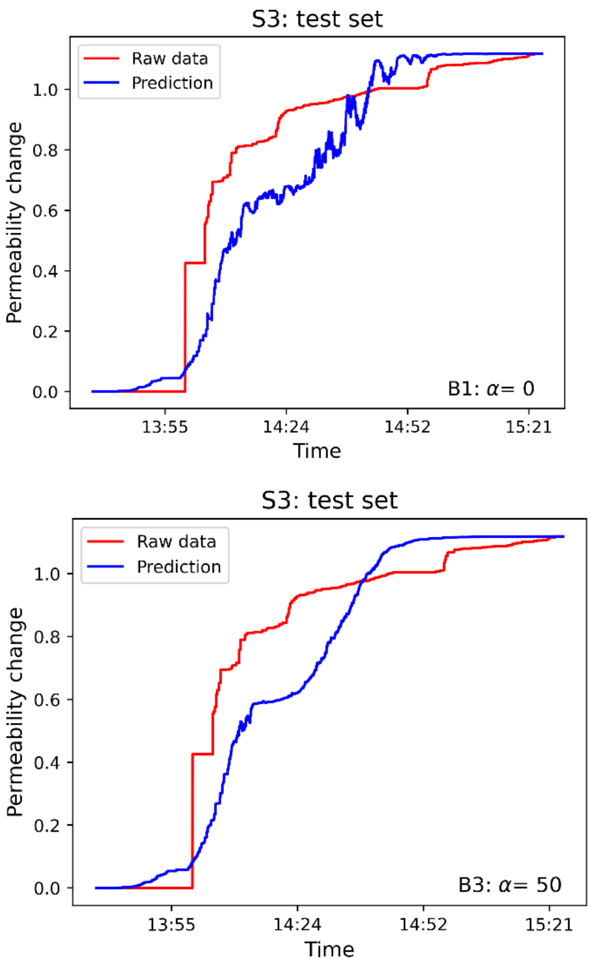

Figure S7: Comparison between raw permeability data (ground truth) and predictions on test set (S3) for Utah FORGE for different α values. With increasing α, the prediction curve on the test set monotonically increases. $\alpha = 150$ is used in this study shown in Fig. 6B of the manuscript.

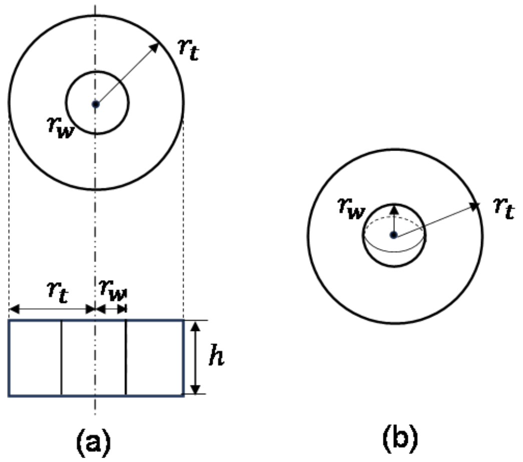

Figure S8: Conceptual flow diagram of radial (a) and spherical (b) steady flow. Here r_w is the wellbore radius, r_t is the radius to the external flow boundary and h is the length of the borehole/cylindrical-zone.

Figure S8 text

Fig. S8(a) illustrates the conceptual steady radial flow regime for constant injection rate, Q at differential pressure, P as:

$$\frac{Q}{2\pi rh} = \frac{k}{\mu}\frac{dP}{dr} \tag{S-1}$$

where r is the distance from the borehole wall, h is the length of the borehole section, k is permeability and μ is dynamic viscosity of the fluid. After variable separation and integration, we obtain:

$$\frac{Q}{2\pi h}\int_{r_w}^{r_t}\frac{dr}{r} = \frac{k}{\mu}\int_{P_e}^{P_d} dP \tag{S-2}$$

with average permeability could be expressed as:

$$k = \frac{\mu Q}{2\pi h(P_d - P_e)}\ln\left(\frac{r_t}{r_w}\right) \tag{S-3}$$

where P_d is the downhole injection pressure. P_e is the far-field pressure at the external radial boundary – defined by the location of the most distant MEQs. The injectivity (I) is the ratio between injection rate and pressure differential ($\Delta P = P_d - P_e$) as $I = Q/\Delta P$, Eq.(S-3) and may be further expressed as:

$$k = \frac{\mu I}{2\pi h}\ln\left(\frac{r_t}{r_w}\right) \tag{S-3}$$

Similarly, for the spherical steady flow shown in Fig.S8(b), the appropriate expression is:

$$\frac{Q}{4\pi R^2} = \frac{k}{\mu}\frac{dp}{dr} \tag{S-4}$$

After variable separation and integration, Eq.(S-4) this becomes:

$$\frac{Q}{4\pi}\int_{r_w}^{r_t}\frac{dr}{r^2} = \frac{k}{\mu}\int_{P_e}^{P_d} dP \tag{S-5}$$

Thus the average permeability for spherical flow may be expressed as:

$$k = \frac{\mu Q}{4\pi(P_d - P_e)}\left(\frac{1}{r_w} - \frac{1}{r_t}\right) \tag{S-6}$$

And substituting injectivity (I) into Eq.(S-6) yields:

$$k = \frac{\mu I}{4\pi}\left(\frac{1}{r_w} - \frac{1}{r_t}\right) \tag{S-7}$$

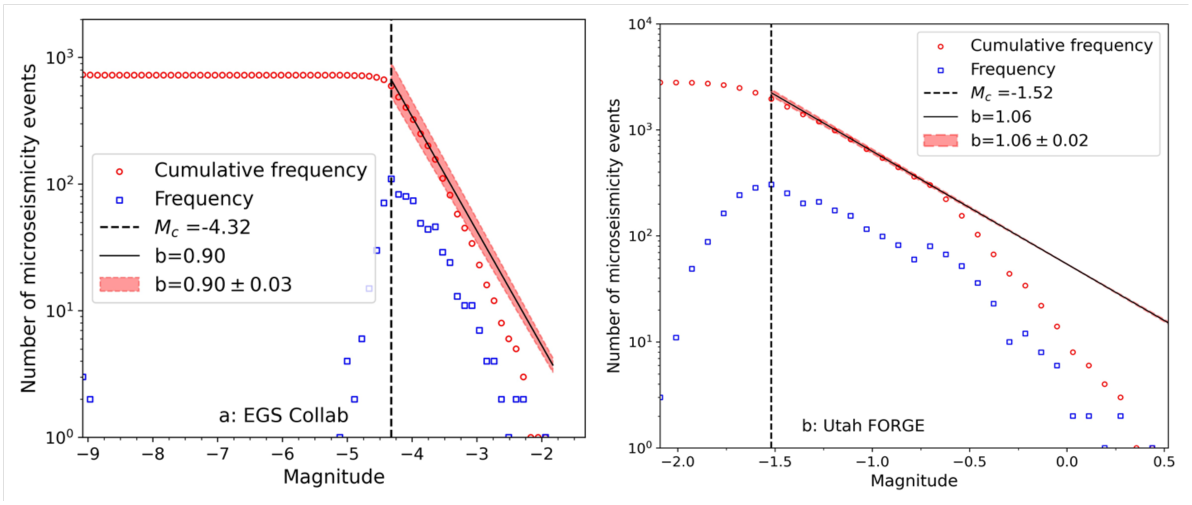

Figure S9: Magnitude frequency distribution and Gutenberg-Richter fit with uncertainty analysis for EGS Collab (a) and Utah FORGE (b) datasets, respectively.

Figure S9 text

High resolution seismic monitoring networks were deployed for the EGS Collab and Utah FORGE stimulations. For detailed information regarding the monitoring system, processing, quality control and quality evaluation of the seismic catalogs and statistical parameters, please refer to Schoenball et al. (2020) and Rutledge et al. (2021). We analysed the magnitude of completeness (M_c) and b-values for the EGS Collab and Utah FORGE MEQ catalogs, as shown in Fig.S9. The maximum curvature method (Wiemer & Wyss, 2002) was used to calculate M_c, which is the maximum value of the first derivative of the frequency magnitude curve. The b-value is calculated by the maximum likelihood method (Aki, 1965). We also completed uncertainty analysis for the b-value by calculating the standard error associated with the maximum likelihood method (Shi & Bolt, 1982).

We obtained $M_c = -4.32$ and -1.52 for EGS Collab and Utah FORGE earthquake catalogs, respectively. Both values are quite small, consistent with the capability of the seismic networks to capture even small MEQs. Also, based on the linear proportionality relationship between permeability change and seismic moment (see Discussion and Eqs.17-18), the effect of these extremely small magnitude events on permeability changes will be small – and missing these events is unlikely to impact our analysis or conclusions. The b-value for the EGS Collab and Utah FORGE are $b = 0.90 \pm 0.03$, $b = 1.06 \pm 0.02$ respectively. Based on the Gutenberg-Richter relation, a b-value near 1.0 implies a typical distribution of earthquakes, with smaller events being more common than larger ones.

Table S1: Comparison of various standalone machine learning and deep learning models trained on Utah FORGE. All deep learning models were trained across 10 trials, with average R^2 scores and variances reported across data splits.

Models (Utah FORGE)	Train (S1) R^2	Validation (S2) R^2	Test (S3) R^2
Bi-LSTM	0.99 ± 0.002	0.91 ± 0.005	0.85 ± 0.002
Bi-GRU	0.92 ± 0.001	0.95 ± 0.001	0.81 ± 0.002
Linear Regression	0.7	0.78	0.63
Elastic-Net	0.56	0.67	0.59
XGBoost	1.0	0.88	0.84
Voting Regression (Ensemble method)	0.95	0.89	0.85

Table S2: Comparison of various standalone machine learning and deep learning models trained on EGS Collab. All deep learning models were trained across 10 trials, with average R^2 scores and variances reported across data splits.

Models (EGS Collab)	Train (EP3) R^2	Validation (EP4) R^2	Test (EP5) R^2
Bi-LSTM	0.99 ± 0.003	0.83 ± 0.07	0.93 ± 0.04
Bi-GRU	0.95 ± 0.008	0.89 ± 0.02	0.88 ± 0.05
Linear Regression	0.63	0.60	0.21
Elastic-Net	0.7	0.52	0.31
XGBoost	1.0	0.85	0.77

Voting Regression (Ensemble method)	0.95	0.89	0.80

Table S3: Zero-shot performance for models trained on **EGS Collab and tested on Utah FORGE**. All deep learning models were trained across 10 trials, with average R^2 scores and variances reported across data splits.

Models (EGS Collab→ Utah FORGE)	S1 R^2	S2 R^2	S3 R^2
Bi-LSTM	0.95 ± 0.0002	0.80 ± 0.0001	0.93 ± 0.0003
Bi-GRU	0.95 ± 0.0004	0.89 ± 0.0002	0.90 ± 0.0004

Table S4: Zero-shot performance for models trained on Utah FORGE and tested on EGS Collab. All deep learning models were trained across 10 trials, with average R^2 scores and variances reported across data splits.

Models (Utah FORGE→ EGS Collab)	EP3 R^2	EP4 R^2	EP5 R^2
Bi-LSTM	0.87 ± 0.00001	0.92 ± 0.00001	0.84 ± 0.00002
Bi-GRU	0.89 ± 0.00002	0.91 ± 0.00002	0.80 ± 0.00002

Table S3-S4 text: Model zero-shot performance

We test the zero-shot capability of our model (Chang et al., 2008) across the two datasets. In this setup, the trained model is tested on a new distribution to see how efficiently it can generalize to the unseen distribution, thus testing its true generalization and continual learning capabilities. Assuming an independent and identical distribution (i.i.d) across data the model is trained using a monotonically decreasing activation function that is Lipschitz continuous in Euclidean space. We show that our model successfully captures this phenomenon and achieves a respectable R^2 score; specifically in Supp. Table S3, we report the performance of the model trained on EGS Collab and tested on all 3 Stages of Utah Forge, achieving R^2 scores of 0.95, 0.80, and 0.93 respectively. Similarly, Supp. Table S4 reports the zero-shot performance of the model trained on Utah FORGE and tested on all 3 episodes of EGS Collab.

Table S5: Transfer learning performance for model trained on Utah FORGE and knowledge is transferred on EGS Collab. All deep learning models were trained across 10 trials, with average R^2 scores and variances reported across data splits.

Models (Utah FORGE→ EGS Collab)	Train (EP3) R^2	Validation (EP4) R^2	Test (EP5) R^2
Bi-LSTM	0.95 ± 0.04	0.89 ± 0.03	0.82 ± 0.05
Bi-GRU	0.95 ± 0.009	0.85 ± 0.05	0.83 ± 0.10

Table S6: Transfer learning performance for models trained on EGS Collab and knowledge is transferred on Utah FORGE. All deep learning models were trained across 10 trials, with average R^2 scores and variances reported across data splits.

Models (EGS Collab→ Utah FORGE)	Train (S1) R^2	Validation (S2) R^2	Test (S3) R^2
Bi-LSTM	0.96 ± 0.004	0.85 ± 0.005	0.89 ± 0.005
Bi-GRU	0.97 ± 0.001	0.90 ± 0.002	0.88 ± 0.007

Table S7: Model performance comparison between models using proposed physics inspired loss and standard MSE loss (α = 0) for standalone models trained on Utah FORGE dataset. All deep learning models were trained across 10 trials, with average R^2 scores and variances reported across data splits.

Models	Train (S1) R^2	Validation (S2) R^2	Test (S3) R^2
Bi-LSTM	0.99 ± 0.002	0.91 ± 0.005	0.85 ± 0.002
Bi-LSTM ($\alpha = 0$)	0.99 ± 0.03	0.71 ± 0.201	0.68 ± 0.2
Bi-GRU	0.92 ± 0.001	0.95 ± 0.001	0.81 ± 0.002
Bi-GRU ($\alpha = 0$)	0.92 ± 0.03	0.69 ± 0.217	0.65 ± 0.202

Table S8: Model performance comparison between models using proposed physics inspired loss with standard MSE loss α = 0) for standalone models trained on EGS Collab dataset. All deep learning models were trained across 10 trials, with average R^2 scores and variances reported across data splits.

Models	Train (EP3) R^2	Validation (EP4) R^2	Test (EP5) R^2
Bi-LSTM	0.99 ± 0.003	0.83 ± 0.07	0.93 ± 0.04
Bi-LSTM ($\alpha = 0$)	0.99 ± 0.03	0.73 ± 0.16	0.13 ± 0.03
Bi-GRU	0.95 ± 0.008	0.89 ± 0.02	0.88 ± 0.05
Bi-GRU ($\alpha = 0$)	0.94 ± 0.01	0.65 ± 0.09	0.59 ± 0.13

Table S9 Comparison of Bi-LSTM models using different values of Δt_w trained on EGS Collab. All models are trained across 10 trials, with average R^2 scores and variances reported across data splits.

Δt_w (min)	Train (EP3) R^2	Validation (EP4) R^2	Test (EP5) R^2
2	0.99 ± 0.003	0.83 ± 0.07	0.93 ± 0.04
4	0.99 ± 0.00004	0.42 ± 0.003	0.74 ± 0.017
6	0.99 ± 0.0005	0.14 ± 0.00007	0.27 ± 0.006
8	0.96 ± 0.0015	0.19 ± 0.0002	0.65 ± 0.004
10	0.98 ± 0.0001	0.31 ± 0.0004	0.4 ± 0.06

Table S10 Comparison of Bi-LSTM models using different values of Δt_w trained on Utah FORGE. All models are trained across 10 trials, with average R^2 scores and variances reported across data splits.

Δt_w (min)	Train (EP3) R^2	Validation (EP4) R^2	Test (EP5) R^2
2	0.99 ± 0.002	0.91 ± 0.005	0.85 ± 0.002
4	0.99 ± 0.00003	0.90 ± 0.0001	0.53 ± 0.00005
6	0.99 ± 0.00004	0.90 ± 0.0003	0.48 ± 0.00005
8	0.99 ± 0.000003	0.88 ± 0.0003	0.47 ± 0.0006
10	0.99 ± 0.00003	0.86 ± 0.0004	0.52 ± 0.0006

Table S11: Comparison of LSTM and Bi-LSTM models trained on EGS collab. All models were trained across 10 trials, with average R^2 scores and variances reported on test set.

Model	Test (EP5) R^2	Average Epochs
Bi-LSTM	0.93 ± 0.04	700
LSTM	0.90 ± 0.09	2100

Table S11: Text

We are dealing with a stateful problem, which requires access to autoregressive connections. We selected two widely used RNNs, the Gated Recurrent Unit (GRU) and the Long-term Memory (LSTM) model. We observed that LSTM outperforms GRU in the majority of scenarios. Both uni-directional LSTM and bi-directional LSTM can efficiently model sequential data; however, a bi-model is advantageous when the output depends on the entire predictor sequence as it captures both backward and forward dependencies through time (Goodfellow et al., 2016, Stogin et al., 2020, Mali et al., 2023). In addition, the uni-directional model takes significantly more time to converge and is less stable compared to bi-directional. We compare an average number of epochs required by both models to converge on the EGS collab dataset (Table S11); as evident from the result, the bi-directional model converges much faster and is more stable with smaller standard deviation of R^2 than the uni-directional model.

Table S12: Nomenclature

a	Edge dimension of the area of the transected fault	M_0^s	Seismic energy released for slip
A	Fault patch area where slip occurred as $A \sim a^2$	M_0^n	Seismic energy released during fracture opening or closing
b_o	Original fracture aperture	$\mathcal{M}$	cumulative logarithmic seismic moment
b_s	Shear fracture aperture increment	P	Wellhead pressure
Δb	Fracture aperture change	Q	Flow rate
D	hydraulic diffusivity	r	Separation between the migrating seismic front and injection point
h	borehole/cylindrical-zone length	r_t	Radius to the migrating external flow boundary (defined by MEQ seismic front)
i	Fracture dilation angle	r_w	Injection wellbore radius
I	Injectivity	s	Spacing between adjacent parallel fractures
G	Shear modulus	Δt	elapsed time since initiation of injection t_0
k	Average permeability	Δt_w	Span of moving time window for seismicity rate calculation
k_c	Normalized permeability change	Δu_s	Fracture slip offset
k_0	Initial permeability	Δu_n	Normal displacement in fracture opening or closing

Δk	Permeability change compared to initial permeability	V	Volume of media surrounding the fault destressed by slip
Δk_s	Permeability change resulting from fracture shearing	α	Non-negative scalar penalty coefficient
Δk_n	Permeability change resulting from fracture tensile opening	μ	Fluid viscosity
$\mathcal{L}$	Adjusted loss function	λ	Seismicity rate
M_0	Seismic moment	$\Delta\tau$	Shear stress drop
M_w	Moment magnitude		

References:

[1] Goodfellow, I., Bengio, Y., & Courville, A. (2016). Sequence modeling: recurrent and recursive nets. Deep learning, 367-415.

[2] Stogin, J., Mali, A., & Giles, C. L. (2020). A provably stable neural network Turing Machine. arXiv preprint arXiv:2006.03651.

[3] Mali, A., Ororbia, A., Kifer, D., & Giles, L. (2023). On the Computational Complexity and Formal Hierarchy of Second Order Recurrent Neural Networks. arXiv preprint arXiv:2309.14691.

[4] Schoenball, M., Ajo-Franklin, J. B., Blankenship, D., Chai, C., Chakravarty, A., Dobson, P., ... & EGS Collab Team. (2020). Creation of a mixed-mode fracture network at mesoscale through hydraulic fracturing and shear stimulation. Journal of Geophysical Research: Solid Earth, 125(12), e2020JB019807.

[5] Rutledge, J., Pankow, K., Dyer, B., Wannamaker, P., Meier, P., Bethmann, F., & Moore, J. (2021). Seismic Monitoring at the Utah FORGE EGS Site. GRC Transactions, 45, 12.

[6] Wiemer, Stefan, & Wyss, M. (2002). Mapping spatial variability of the frequency-magnitude distribution of earthquakes. In Advances in geophysics (Vol. 45, pp. 259–V). Elsevier.

[7] Aki, K. (1965). Maximum likelihood estimate of b in the formula log N= a-bM and its confidence limits. Bull. Earthq. Res. Inst., Tokyo Univ., 43, 237–239.

[8] Shi, Yaolin, & Bolt, B. A. (1982). The standard error of the magnitude-frequency b value. Bulletin of the Seismological Society of America, 72(5), 1677–1687. https://doi.org/10.1785/BSSA0720051677
